# Proceedings of Pennsylvania Society of Dental Surgery

**Published:** 1860-03

**Authors:** 


### PROCEEDINGS OF PENNSYLVANIA SOCIETY OF DENTAL SURGERY.

MESSRS. EDITORS:—There was a profitable meeting of this Association held last evening, a brief report of which I offer you in place of, and of more interest than, my usual letter.

Dr. McQUILLEN offered a very interesting discussion on the subject of the absorption of the fangs of the deciduous teeth, his theory being, a diminution or entire withdrawal of the suply of nutrition, occasioned by the demand for and in the growth of the permanent teeth. He illustrated by copious references to the law of waste and supply in the animal economy, and of the cessation of a function when no longer required. The argument was able and comprehensive, and I trust will be given in *extenso* in some of our journals.

Other gentlemen spoke to the question, some contending that absorbtion was due to *pressure* as well as defective nutrition, and citing cases where the deciduous teeth had remained and were useful in the absence of the permanent teeth, they never having been developed. After a pretty full discussion of this interesting topic

Dr. J. M. HARRIS introduced the subject of "*accidents in dental practice—how to meet and treat them*," in which he enumerated Luxation of the lower jaw—Syncope—the propriety of operating for females in a delicate condition—Fractures and Hemorrhage.

Of the first topic, numerous and some humorous examples were given,—the fright of the operator with his first case and his bungling attempts to reduce it, to the imminent danger of the mutilation of his thumbs, were ludicrous in the extreme. The usual means employed to reduce luxation, and the necessity for supporting the lower jaw while operating for such persons as were pre-disposed to such accidents, were alluded to, also the causes for such tendency, etc., when the second topic, *Synocope*, received attention, and the ordinary treatment,—the administration of a few drops of aqua ammonia in water and a recumbent posture, with plenty of fresh air— was suggested; and the too prevalent habit of passing strong ammonia under the nose and dashing cold water in the face, very properly condemned as not only useless but sometimes dangerous.

On the topic of the treatment of females when in a certain condition, there was some difference of opinion, some contending that the extraction of a painful tooth is less hurtful than the continual suffering from tooth-ache, while others recommended palliatives, such as protecting the offending tooth by a cap placed upon an adjoining tooth, thus preventing contact on the occlusion of the jaws; and such other temporizing measures as are ordinarily resorted to in such cases.

*Fractures* was next considered. Fracture of the alveolus was thought a trifling matter but that it was important to remove all detached bone and other substance.

Dr. FITCH here cited a case of the fracture of the jaw and gave his treatment—the employment of a platina plate, adapted as well as was possible under the circumstances, with fine soft platina wire attachments to various teeth, thus drawing the fractured ends in position, and by which a cure was affected.

During the discussion on Hemorrhage, Dr. Garretson (who treated this subject as well as others, at considerable length) alluded to the employment of *cobwebs*,—which he had suggested some years ago in a communication to the *Dental News Letter*,—combined with some powerful astringent and styptic, as being an admirable remedy, also other remedies, such as a preparation of iron, the actual cautery, etc., and that if all failed and syncope ensued, that then the hemorrhage would generally be arrested as a sequence of fainting.

I attempt, in the above, but a mere synopsis of the discussions, and which is penned from memory alone, therefore my excuse for any imperfections. Yours, O. U. C.

PHILA., Feb. 15, 1860.

